# Supplementary material for: Segmentation of Dental Restorations on Panoramic Radiographs Using Deep Learning
Source: Diagnostics (Basel). 2022 May 25;12(6):1316. doi: 10.3390/diagnostics12061316 (PMC9221749; doi:10.3390/diagnostics12061316)
Supplement: Supplementary file 1 [file diagnostics-12-01316-s001.zip › diagnostics-1725290-supplementary.pdf]

diagnostics

Supplementary Materials

Segmentation of Dental Restorations on Panoramic Radiographs Using Deep Learning

Csaba Rohrer[1], Joachim Krois[1,2], Jay Patel[3], Hendrik Meyer-Lueckel[4], Jonas Almeida Rodrigues[1,5], Falk Schwendicke[1,2]

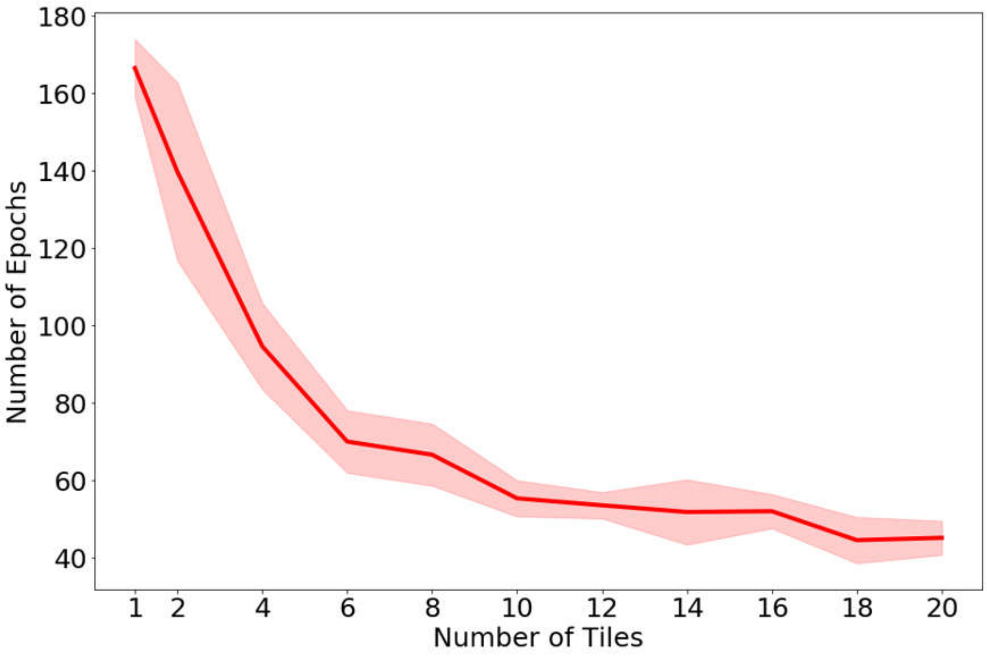

Figure S1. Conversion of model training depending on the number of tiles.

Table S1. Results for different restorations and tile numbers.

			**Fillings**		
**Tiles**	Epochs	IoU	Precision	Sensitivity	F-Score
**1**	166	0.48	0.85	0.56	0.57
**2**	139	0.68	0.85	0.78	0.77
**4**	94	0.71	0.87	0.80	0.78
**6**	70	0.76	0.90	0.83	0.81
**8**	66	0.89	0.95	0.93	0.92
**10**	55	0.93	0.97	0.95	0.95
**12**	53	0.93	0.97	0.95	0.95
**14**	51	0.91	0.96	0.94	0.93
**16**	52	0.92	0.97	0.95	0.94
**18**	44	0.92	0.96	0.94	0.94

**20**	45	0.93	0.97	0.95	0.94
			**Root-canal Fillings**		
**Tiles**	Epochs	IoU	Precision	Sensitivity	F-Score
**1**	166	0.33	0.97	0.33	0.33
**2**	139	0.55	0.97	0.57	0.57
**4**	94	0.74	0.95	0.77	0.77
**6**	70	0.84	0.97	0.86	0.86
**8**	66	0.86	0.97	0.88	0.88
**10**	55	0.91	0.98	0.93	0.93
**12**	53	0.92	0.98	0.94	0.94
**14**	51	0.95	0.98	0.96	0.96
**16**	52	0.95	0.98	0.97	0.97
**18**	44	0.96	0.98	0.97	0.97
**20**	45	0.96	0.98	0.97	0.97
			**Crowns**		
**Tiles**	Epochs	IoU	Precision	Sensitivity	F-Score
**1**	166	0.85	0.91	0.93	0.89
**2**	139	0.89	0.94	0.94	0.92
**4**	94	0.87	0.95	0.92	0.90
**6**	70	0.88	0.95	0.92	0.90
**8**	66	0.94	0.98	0.96	0.95
**10**	55	0.97	0.99	0.98	0.97
**12**	53	0.96	0.99	0.98	0.97
**14**	51	0.96	0.98	0.97	0.97
**16**	52	0.97	0.99	0.98	0.97
**18**	44	0.96	0.98	0.97	0.97
**20**	45	0.96	0.98	0.98	0.97
			**Implants**		
**Tiles**	Epochs	IoU	Precision	Sensitivity	F-Score
**1**	166	0.90	0.99	0.91	0.90
**2**	139	0.97	0.99	0.98	0.98
**4**	94	0.98	0.98	0.99	0.98
**6**	70	0.98	0.99	0.99	0.99
**8**	66	0.98	0.99	0.99	0.98
**10**	55	0.99	1.00	0.99	0.99
**12**	53	0.99	1.00	0.99	0.99
**14**	51	0.99	1.00	1.00	0.99
**16**	52	0.99	1.00	1.00	0.99
**18**	44	0.99	1.00	1.00	0.99
**20**	45	0.99	1.00	1.00	0.99
			**Overall**		
**Tiles**	Epochs	IoU	Precision	Sensitivity	F-Score

**1**	166	0.59	0.87	0.64	0.70
**2**	139	0.73	0.89	0.79	0.82
**4**	94	0.74	0.89	0.80	0.82
**6**	70	0.76	0.91	0.81	0.83
**8**	66	0.84	0.95	0.87	0.88
**10**	55	0.90	0.97	0.92	0.92
**12**	53	0.90	0.97	0.93	0.93
**14**	51	0.91	0.97	0.94	0.94
**16**	52	0.92	0.97	0.94	0.95
**18**	44	0.92	0.97	0.94	0.95
**20**	45	0.93	0.97	0.95	0.95
